# Unraveling genetic causality between metformin and myocardial infarction on the basis of Mendelian randomization

**DOI:** 10.3389/fendo.2024.1376464

**Published:** 2024-05-03

**Authors:** Yongru Zhuang, Xiaojun Pan, Ya Chen, Jinfang Song

**Affiliations:** ^1^ Jiangsu Key Laboratory of New Drug Research and Clinical Pharmacy, Xuzhou Medical University, Xuzhou, China; ^2^ Department of Pharmacy, Affiliated Hospital of Jiangnan University, Wuxi, China; ^3^ Department of Pharmacy, Wuxi No.5 People’s Hospital, Wuxi, China; ^4^ Department of Endocrinology, Affiliated Hospital of Jiangnan University, Wuxi, China

**Keywords:** metformin, myocardial infarction, cardiovascular disease, diabetes, Mendelian randomization study

## Abstract

**Background:**

In recent years, several studies have explored the effect of metformin on myocardial infarction (MI), but whether metformin has an improvement effect in patients with MI is controversial. This study was aimed to investigate the causal relationship between metformin and MI using Mendelian randomization (MR) analysis.

**Methods:**

The genome-wide significant (*P*<5×10^-8^) single-nucleotide polymorphisms (SNPs) in patients with metformin and patients with MI were screened from the Open genome-wide association study (GWAS) project as instrumental variables (IVs). The study outcomes mainly included MI, old MI, acute MI, acute transmural MI of inferior wall, and acute transmural MI of anterior wall. The inverse variance weighted (IVW) method was applied to assess the main causal effect, and weighted median, simple mode, weighted mode methods, and MR-Egger regression were auxiliary applied for supplementary proof. The causal relationship between metformin and MI was assessed using odds ratios (OR) and 95% confidence intervals (95% CI). A leave-one-out method was used to explore the effect of individual SNPs on the results of IVW analyses, and a funnel plot was used to analyze the potential bias of the study results, thus ensuring the robustness of the results.

**Results:**

In total, 16, 84, 39, 26, and 34 SNPs were selected as IVs to assess the genetic association between metformin and outcomes of MI, old MI, acute MI, acute transmural MI of inferior wall, and acute transmural MI of anterior wall, respectively. Treatment with metformin does not affect the risk of acute transmural MI of anterior wall at the genetic level (*P*>0.05; OR for inverse variance weighted was 1.010). In the cases of MI, old MI, acute MI, and acute transmural MI of inferior wall, metformin may even be a risk factor for patients (*P*<0.05; ORs for inverse variance weighted were 1.078, 1.026, 1.022 and 1.018 respectively). There was no horizontal pleiotropy or heterogeneity among IVs. The results were stable when removing the SNPs one by one.

**Conclusion:**

Metformin is not protective against the risk of myocardial infarction in patients and may even be a risk factor for MI, old MI, acute MI, and acute transmural MI of inferior wall.

## Background

Mortality and disability rates caused by cardiovascular disease (CVD) are very high worldwide (1), more than twice the mortality rate of cancer, creating a severe burden on global public health (2). In 2019, there were approximately 18.6 million cardiovascular deaths globally, of which 1,080 occurred in Asia, accounting for 35% of the total deaths in Asia (3). According to reports published by the American Heart Association, CVD is the leading cause of death in the United States (4). As an important pathogenesis factor of CVD, type 2 diabetes mellitus (T2DM) causes a variety of large vascular diseases such as coronary heart disease and cerebrovascular diseases and microvascular complications such as diabetic nephropathy and retinopathy because of its insulin resistance (5, 6). CVD has become the main cause of death in T2DM patients. In addition, risk factors for CVD include myocardial infarction (MI), stroke, hypertension, dyslipidemia and so on (7), among which more than half of cardiovascular deaths are caused by acute myocardial infarction (AMI) (8). Therefore, the discovery of effective drugs to treat MI and improve its prognosis is critical to reducing cardiovascular mortality and improving global health. In recent years, researchers continue to explore drugs to improve the prognosis of MI, among which metformin, a drug used in the clinical treatment of T2DM, has attracted great attention.

Metformin is recommended as the basic drug for T2DM treatment by most national guidelines, including the guidelines of the American Diabetes Association, the European Association for the Study of Diabetes and the National Institute for Health and Care Research of the United Kingdom (9). Metformin mainly plays a hypoglycemic role by activating adenosine monophosphate activated protein kinase (AMPK) in cells and reducing glucose output from the liver (10). In addition to hypoglycemic effects, metformin also has many other effects including anti-cancer (11, 12), anti-inflammatory (13, 14) and anti-aging (15, 16). Currently, pharmacogenomic studies of metformin focus on genes such as organic cation transporters (OCTs), plasma membrane monoamine transporter (PMAT) and multidrug and toxic compound extrusions (MATEs) affecting its pharmacokinetics and AMPK, ataxia telangiectasia-mutated (ATM), glucose transporter type (GLUT2) and carboxypeptidase A6 (CPA6) affecting its pharmacodynamics, most of these studies have explored the influence of gene polymorphism on the hypoglycemic effect of metformin. However, the effect of the single-nucleotide polymorphisms (SNPs) on the cardiovascular protective effect of metformin has not been reported (17). Several studies have shown that metformin may have certain cardiovascular benefits for both diabetic and non-diabetic patients (18, 19), but most clinical trials are small in scale, and whether metformin is beneficial for patients with MI remains doubtful. In a 10-year follow-up study of diabetic patients, the risk of MI in diabetic patients taking metformin was significantly reduced (20). Another cohort study found that the use of metformin in T2DM patients increases the risk of cardiovascular disease death during the first occurrence of AMI, while taking metformin after stable MI may have a protective effect (21). The above researches indicate that metformin has a positive effect on improving the outcome in diabetic patients with MI, but this effect does not exclude the benefit of metformin on blood glucose control. While exploring the protective mechanisms of metformin against MI beyond its hypoglycemic effect, Wang M et al. found that metformin reduced MI size in mice by inhibiting Heat shock factor 1 (HSF1) (22). Moreover, some researchers have confirmed that metformin can indeed reduce the fibrosis and inflammation in the hearts of mice after MI (23). In addition, a retrospective study showed that long-term metformin treatment reduced the size of MI (24), which seems to indicate that metformin does have a role to reduce the risk of MI in patients. However, another study showed that no statistically significant cardioprotective correlation was found between metformin and MI size in patients with diabetes and acute ST elevation MI (25). Hartman et al. collected two-year follow-up data after metformin treatment for 4 months in 379 patients with MI without diabetes after PCI. It was found that 4 months of metformin treatment did not reduce the incidence of cardiovascular events compared with placebo (26), which was consistent with the conclusion of a randomized controlled trial conducted by Goldberg (27). One meta-analysis found that combination therapy with metformin may even increase the risk of cardiovascular mortality (28). Therefore, current studies have shown that whether metformin can improve MI is still controversial (29, 30), and the relationship needs to be further explored.

Mendelian randomization (MR) analysis is an emerging epidemiological approach that uses comprehensive statistics from genome-wide association studies (GWAS) to infer causal relationships between certain diseases and exposure factors to identify potential risk factors. The instrumental variable in MR analysis is the SNP, which uses the known association between SNP and particular trait or disease to randomly group individuals according to their genotype to infer a causal relationship between the SNP and the disease or trait. By using genetic variation as an instrumental variable for exposure factors, MR analysis can overcome common confounding factors in observational studies (31). In this study, the principle of MR was applied to explore the causal relationship between the therapeutic effect of metformin on MI in order to further explore the novel pharmacological effects of metformin and provide alternative therapeutic drugs for patients with MI.

## Methods

### Study design

**Figure 1:**
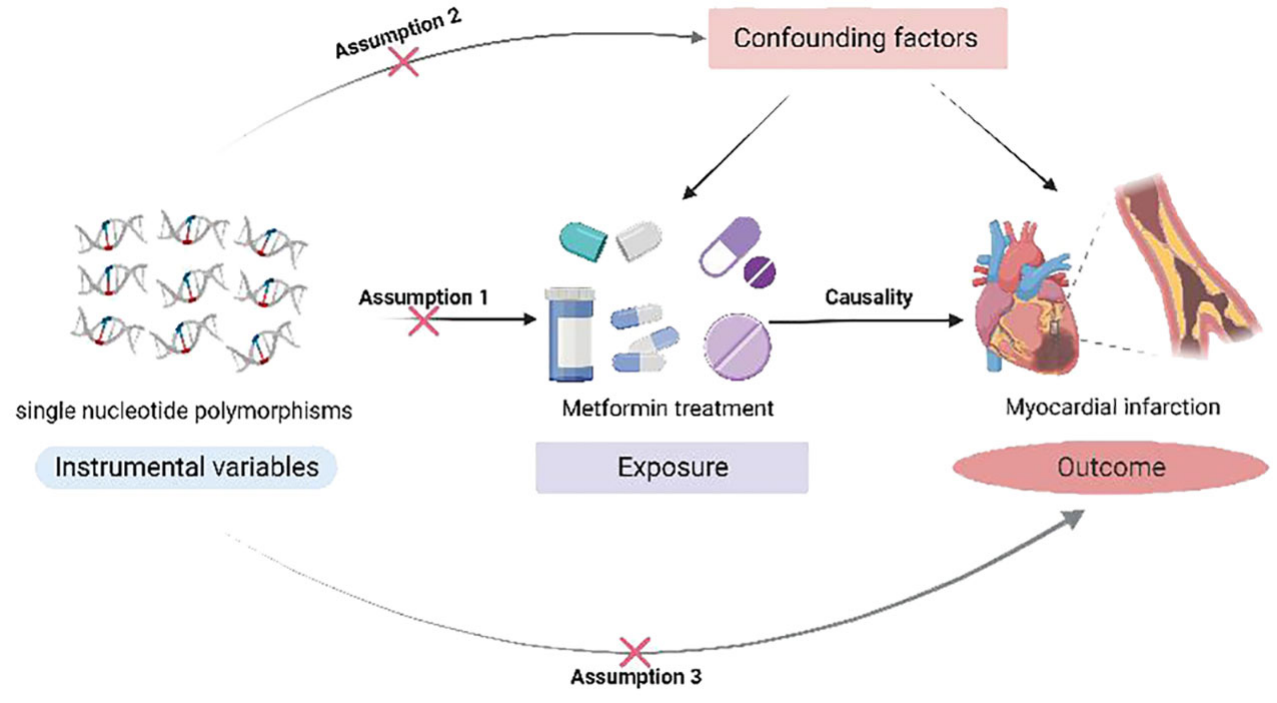
A Mendelian randomization study revealing causality between metformin and myocardial infarction.

In this study, SNPs associated with metformin was used as instrumental variables (IVs) to explore the causal relationship between metformin administration and MI using two-sample MR analysis based on the Open GWAS project. IVs need to satisfy three core assumptions (32): (1) hypothesis of correlation: genetic variation is associated with metformin use. (2) hypothesis of independence: genetic variation is not associated with confounding factors affecting exposure and outcome. (3) hypothesis of exclusivity: genetic variation can only affect the outcome variables through exposure. Since the data used in this study were taken from public database, dedicated research ethics approval is unnecessary. The study design is shown in **Figure 1**.

### Data source

The genetic variation data used in this study were obtained from the Open GWAS project (33). The GWAS ID for metformin is ukb-b-14609, as designated in the National Human Genome Research Institute and European Bioinformatics Institute’s (NHGRI-EBI) GWAS catalog (34). Data for MI, Old MI, acute MI, acute transmural MI of anterior wall and acute transmural MI of inferior wall were obtained from the Open GWAS project named ukb-d-19, ukb-b-16662, ukb-b-3469, ukb-b-453 and ukb-b-5126 respectively. The population in the above datasets was the European population, including males and females. The essential information of the dataset is summarized in **Table 1**.

**Table 1:** The essential information of the dataset.

Project name	Dataset	Consortium	Sample size	N case	Population	Year
Metformin	ukb-b-14609	MRC-IEU	462933	11552	European	2018
MI	ukb-d-19	NA	361194	7018	European	2018
Old MI	ukb-b-16662	MRC-IEU	463010	3340	European	2018
Acute MI	ukb-b-3469	MRC-IEU	463010	2321	European	2018
Acute transmural MI of anterior wall	ukb-b-453	MRC-IEU	463010	1294	European	2018
Acute transmural MI of inferior wall	ukb-b-5126	MRC-IEU	463010	1673	European	2018

MI, myocardial infarction; MRC-IEU, medical research council integrative epidemiology unit.

### IVs selection

We selected IVs at the genome-wide significance level (*P*<5.0×10^-8^) (35). To obtain site-independent IVs, we used the “Two Sample MR” package to set the linkage disequilibrium (LD) threshold to R^2^<0.001 and the clump distance to 10,000 kb from 1000 genomic EUR data.

### Statistical analysis

**Figure 2:**
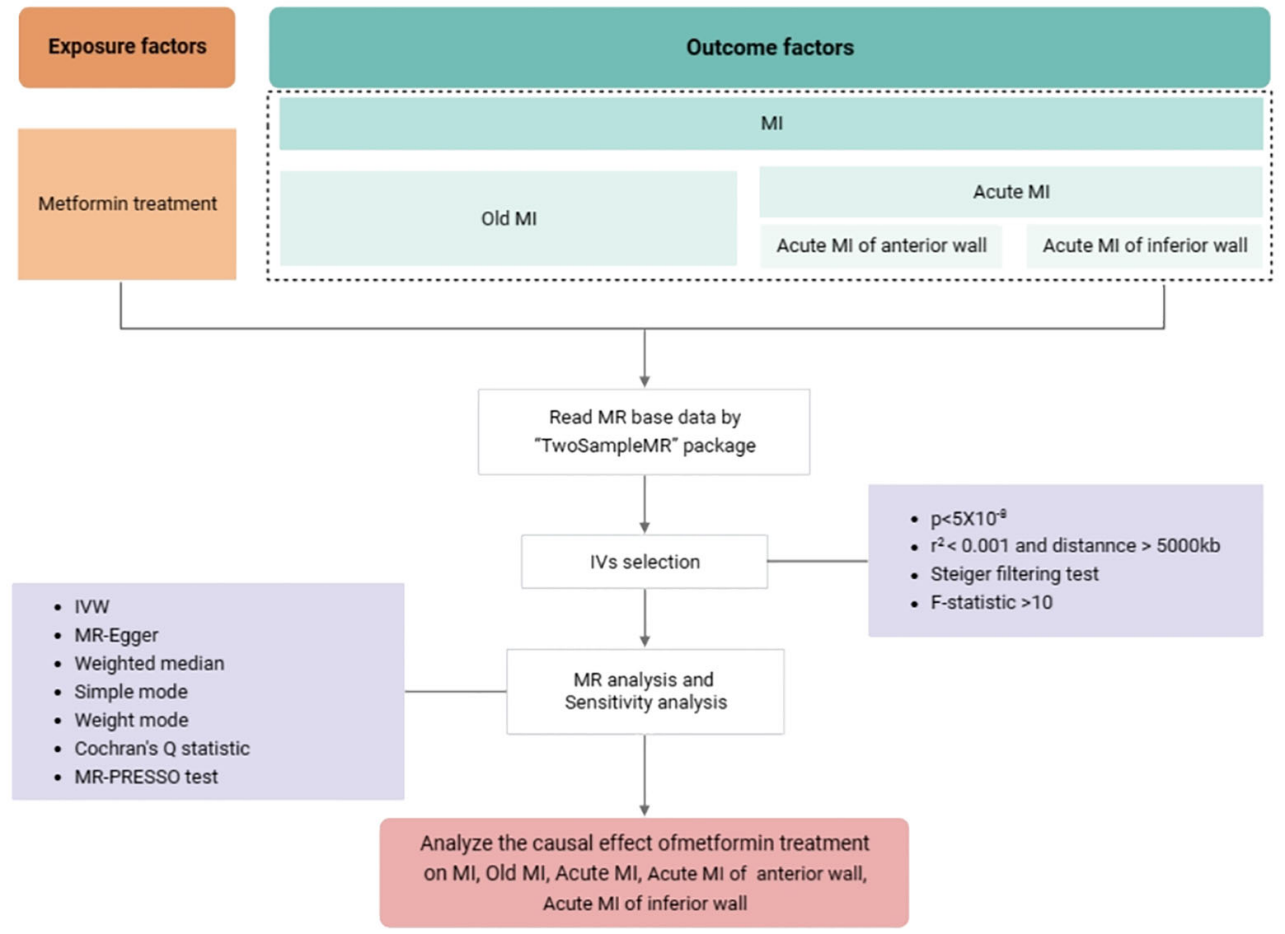
The statistical analysis workflow of the study. Abbreviations used: GWAS: genome-wide association study; IVs, instrumental variables; IVW, inverse variance-weighted; MR, Mendelian randomization; MR-PRESSO, Mendelian randomization pleiotropy residual sum and outlier; MI, myocardial infarction.

The statistical analysis workflow of the study is presented in **Figure 2**. The inverse variance weighted (IVW) method was applied to assess the main causal effects, with the auxiliary application of weighted median, simple mode, weighted mode methods, and MR-Egger regression used for additional supporting evidence. The odds ratio (OR) and 95% confidence interval (CI) value was calculated accordingly. A *P*-value < 0.05 was considered statistically significant. Cochran’s Q test was used to analyze the heterogeneity of IVs (36). If *P*>0.05 then it represents no significant heterogeneity. In MR-Egger regression, if the intercept tends to 0, it can be assumed that there is no horizontal pleiotropy. Where MR-PRESSO global test was used to detect pleiotropy (*P* < 0.05) (37). A leave-one-out method was used to explore the effect of individual SNPs on the results of IVW analyses, and a funnel plot was used to analyze the potential bias of the study results, thus ensuring the robustness of the results (38). All tests were two sided and performed using the R package TwoSampleMR version 0.5.6 in R software 4.2.1.

## Results

### Acquisition of IVs

**Figure 3:**
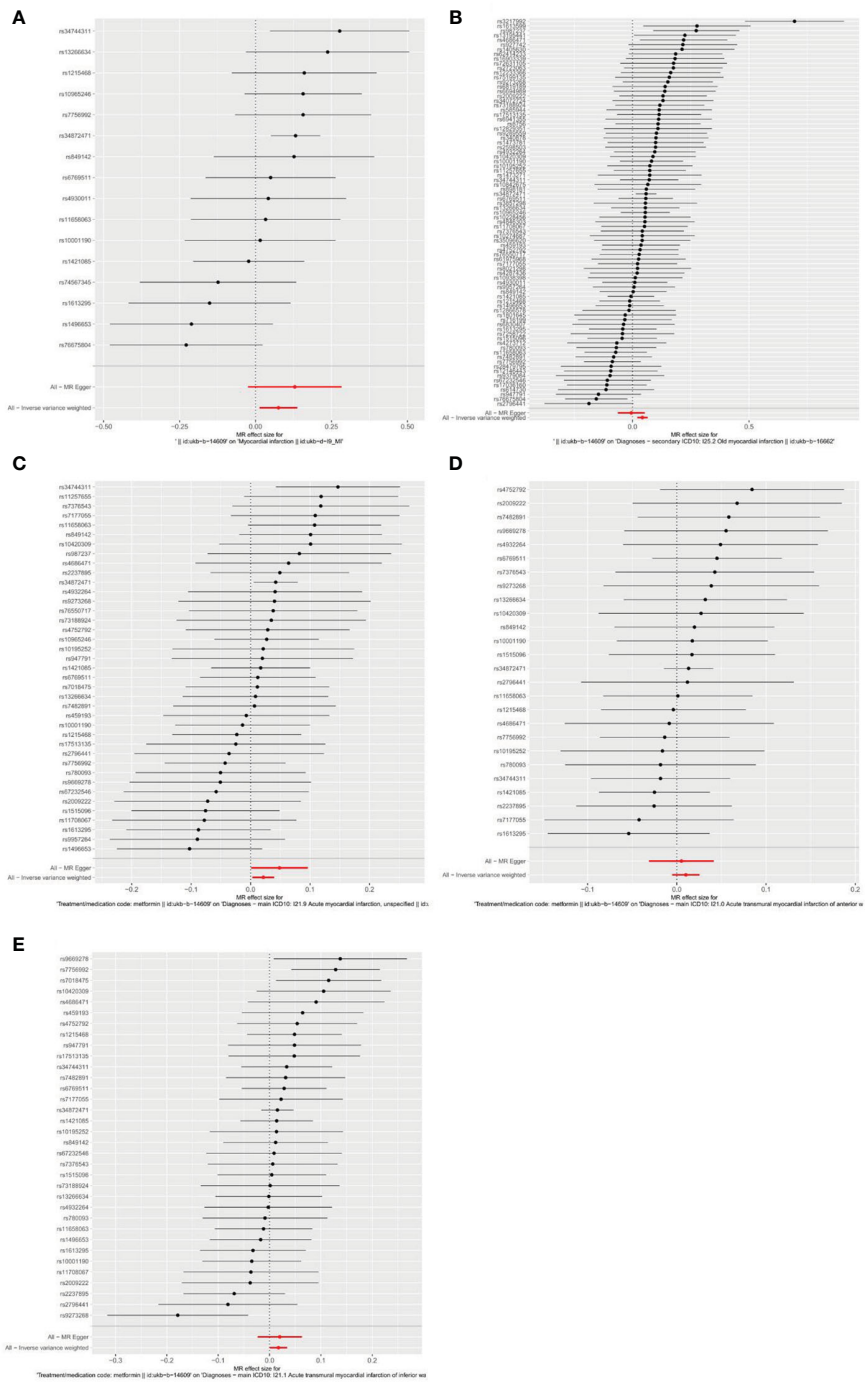
Forest plots of the effect of each SNP on outcomes. **(A)** outcome of myocardial infarction; **(B)** outcome of old myocardial infarction; **(C)** outcome of acute myocardial infarction; **(D)** outcome of acute transmural myocardial infarction of anterior wall; **(E)** outcome of acute transmural myocardial infarction of inferior wall. The black line represents the effects produced by a single SNP, and the red line shows the causal estimate using all instrumental variables. If the solid line is completely to the left of 0, the result estimated by this SNP is that metformin can reduce the risk of outcomes. If the solid line is completely to the right of 0, the result estimated by this SNP is that metformin can increase the risk of outcomes. The result is not significant if the solid line crosses 0.

Firstly, relevant SNPs were obtained through the screening of IVS, and SNPS associated with confounders of MI were removed through the PhenoScanner database search. Meanwhile, palindromic sequences with intermediate allelic frequency were removed during statistical analysis. In total, the metformin GWAS dataset contains 9,851,867 SNPs. Based on the above screening criteria, 16, 84, 39, 26, and 34 SNPs were identified as IVs to assess the genetic association between metformin and outcomes of MI, old MI, acute MI, acute transmural MI of inferior wall, and acute transmural MI of anterior wall, respectively. Detailed information about SNPs is provided in **Supplementary Tables S1**–**S5**. The effect of each SNP on outcomes is displayed in **Figure 3**.

### Causal relationship between metformin and myocardial infarction

**Figure 4:**
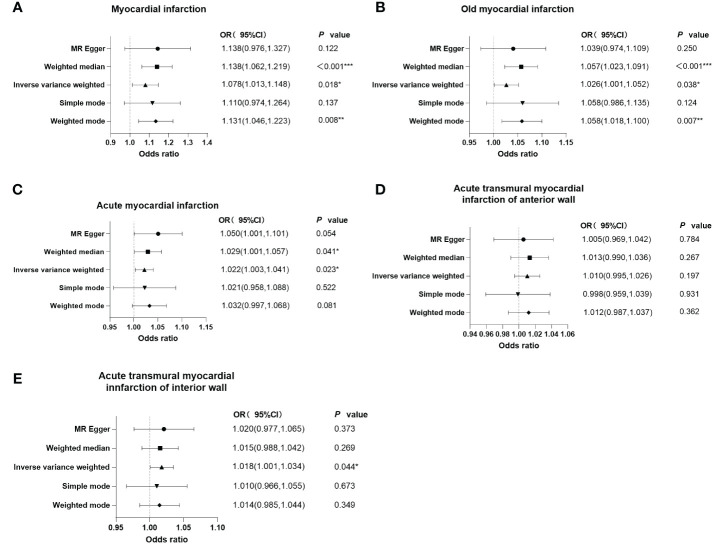
Forest plots of the results of MR-Egger regression, weighted median, inverse variance weighted, simple mode, and weighted mode analysis of metformin on outcomes. **(A)** outcome of myocardial infarction; **(B)** outcome of old myocardial infarction; **(C)** outcome of acute myocardial infarction; **(D)** outcome of acute transmural myocardial infarction of anterior wall; **(E)** outcome of acute transmural myocardial infarction of inferior wall. If the solid line is completely to the left of 1, the result estimated by this SNP is that metformin can reduce the risk of outcomes. If the solid line is completely to the right of 1, the result estimated by this SNP is that metformin can increase the risk of outcomes. The result is not significant if the solid line crosses 1.

**Figure 5:**
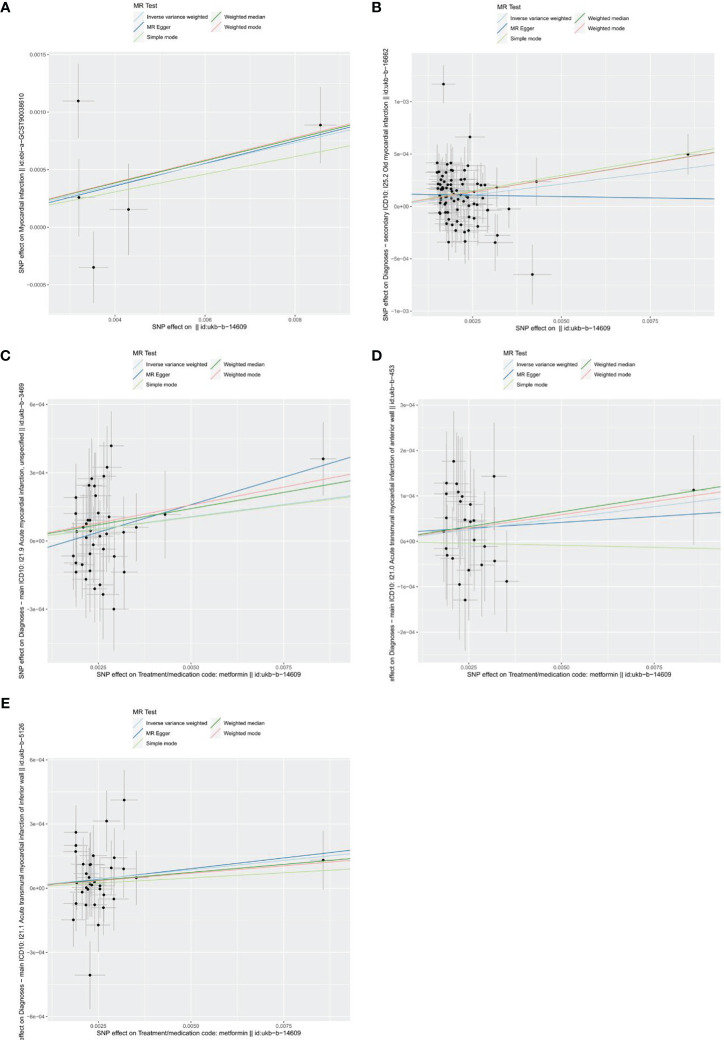
Scatters plots of the effect of metformin on outcomes. **(A)** exposure metformin and outcome myocardial infarction; **(B)** exposure metformin and outcome old myocardial infarction; **(C)** exposure metformin and outcome acute myocardial infarction; **(D)** exposure metformin and outcome acute transmural myocardial infarction of anterior wall; **(E)** exposure metformin and outcome acute transmural myocardial infarction of inferior wall. The black points represent instrumental variables. The horizontal axis represents the effect of SNPs on exposure (metformin). The vertical axis represents the effect of SNPs on the outcomes. Colored lines represent the results of MR analysis based on five methods.

As shown in the forest plots (**Figure 4**), patients treated with metformin had a higher risk of MI (OR=1.078 (1.013-1.148), *P*=0.018), old MI (OR=1.026 (1.001-1.052), *P*=0.038), acute MI (OR=1.022 (1.003-1.041), *P*=0.023), and acute transmural MI of inferior wall (OR=1.018 (1.001 -1.034), *P*=0.044), but there was no significant change in the risk for acute MI infarction of anterior wall (OR=1.010 (0.995 -1.026), *P*=0.197). The scatter plots (**Figure 5**) also showed the same variation in the risk of increased risk of MI in patients treated with metformin.

### Heterogeneity and multiplicity analysis

**Figure 6:**
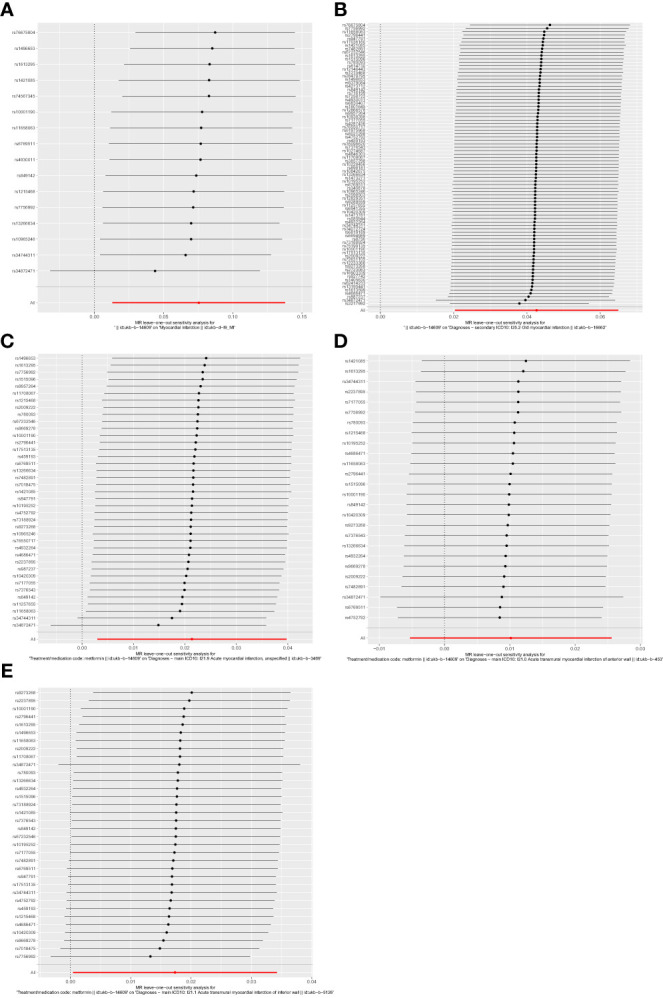
Forest plots of leave-one-out analysis. **(A)** outcome of myocardial infarction; **(B)** outcome of old myocardial infarction; **(C)** outcome of acute myocardial infarction; **(D)** outcome of acute transmural myocardial infarction of anterior wall; **(E)** outcome of acute transmural myocardial infarction of inferior wall. The positions of the red points are greater than zero. The black dots are positioned on the right side of the invalid line. This indicates that removing any of the SNPs will not significantly impact the results.

There was no significant heterogeneity among the IVs by Cochran’s Q test *(P*>0.05). In terms of pleiotropy, MR-Egger regression showed that the intercept of each group was close to 0, and *P*>0.05. MR-PRESSO global test showed *P* > 0.05, which indicated there were no included SNPs found to have potential pleiotropy or outliers on MI, old MI, acute MI, acute transmural MI of inferior wall, or acute transmural MI of anterior wall (**Table 2**). Sensitivity analysis using the leave-one-out method showed that the results were stable when removing the SNPs one by one (**Figure 6**).

**Table 2:** Heterogeneity and multiplicity analysis of metformin and outcomes.

Exposure	Outcome	Method	Q	Q *P* value	egger_intercept	*P* value	MR-PRESSO *P* value
Metformin	MI	MR Egger	23.67	0.050	-2.15550e-04	0.464	0.075
		IVW	24.63	0.055			
	Old MI	MR Egger	52.60	0.072	-3.88839e-05	0.68	0.09
		IVW	52.84	0.083			
	Acute MI	MR Egger	37.90	0.428	-8.28776e-05	0.235	0.409
		IVW	39.40	0.407			
	Acute transmural MI of anterior wall	MR Egger	13.00	0.966	1.59961e-05	0.768	0.970
		IVW	13.09	0.975			
	Acute transmural MI of anterior wall	MR Egger	35.72	0.298	-7.95372e-06	0.898	0.395
		IVW	35.74	0.341			

MI, myocardial infarction; IVW, inverse variance-weighted; MR, Mendelian randomization; MR-PRESSO, Mendelian randomization pleiotropy residual sum and outlier.

### Analysis of bias

**Figure 7:**
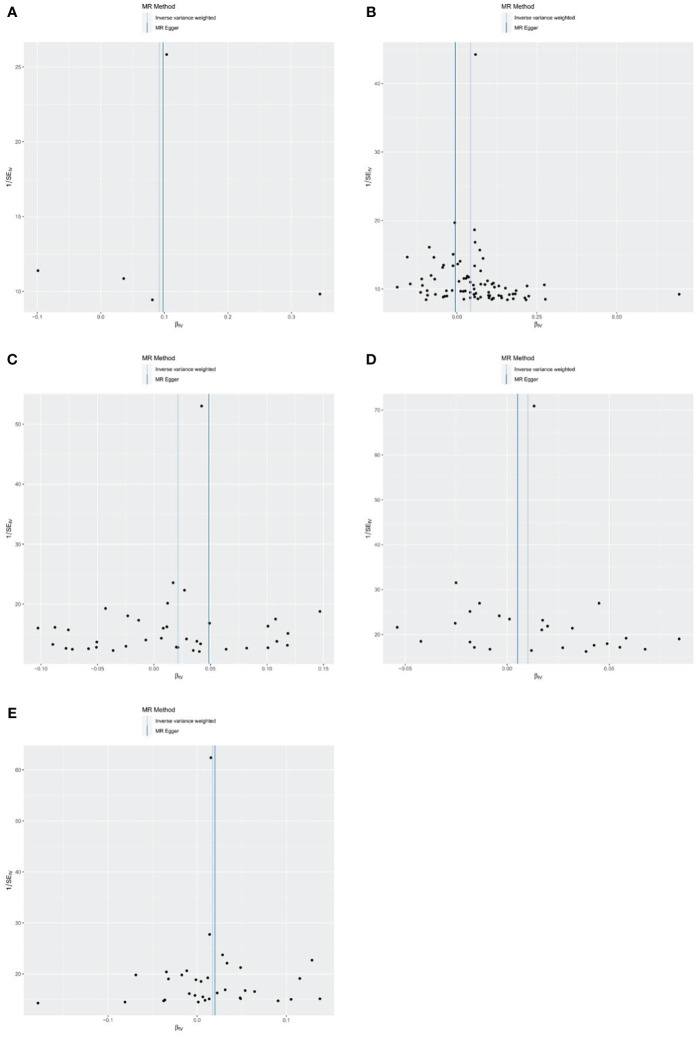
Funnel plots of the causal effect of metformin treatment on outcomes. **(A)** metformin and myocardial infarction; **(B)** metformin and old myocardial infarction; **(C)** metformin and acute myocardial infarction; **(D)** metformin and acute transmural myocardial infarction of anterior wall; **(E)** metformin and acute transmural myocardial infarction of inferior wall. Black points represent SNPs, and the distribution of points is symmetric about the inverse variance weighted and MR-Egger line.

The results of the funnel plots analysis showed basic symmetry and there was no obvious bias on the impact of the results, so the robustness of the analysis results was excellent and the results were stable (**Figure 7**).

## Discussion

Diabetes is a risk factor for cardiovascular death (39). As a first-line antidiabetic agent, metformin mainly plays a hypoglycemic role by activating adenosine monophosphate activated protein kinase (AMPK) in cells and reducing glucose output from the liver. Moreover, its activation of AMPK could reduce cardiomyocyte apoptosis and the formation of myocardial AGEs by enhancing the expression of carnitine palmitoyl transferase 1, thus improving the mitochondrial β -oxidation of the fatty acids and benefiting patients with heart failure (40). When further exploring the cardiovascular protective effect of metformin, the researchers found that metformin may have a potential protective effect of atherosclerotic cardiovascular disease due to its effects on lowering blood glucose, improving endothelial dysfunction, regulating blood coagulation, reducing inflammation and regulating intestinal flora. The possible targets of metformin to impact cardiovascular outcomes in patients include liver kinase B1 (LKB1), AMPK, endothelial nitric oxide synthase (eNOS), phosphatidylinositol 3 kinase-protein kinase B (PI3K-Akt), krüppel-like factor 4 (KLF4), nuclear factor-kappa B (NF-κB) and so on (41). However, it remains unclear whether these effects are beneficial. A clinical prospective study conducted by Sardu et al. found that prediabetic patients increase the burden of inflammation in the adipose tissue around coronary arteries (30). Metformin can improve the prognosis of patients with prediabetic AMI by reducing the inflammatory tension in the pericoronary fat and the ratio of leptin to adiponectin. Another cohort study suggested that use of metformin at the first episode of AMI increases the risk of cardiovascular disease and death in patients with T2DM, and that metformin use after AMI may be beneficial. The above studies suggested that metformin may have an effect on improving the outcome of cardiovascular events in diabetic patients with AMI, and is associated with the process of the development of AMI (21). However, for non-diabetic patients, studies have demonstrated that taking metformin does not improve the prognostic outcome of MI (26). In a randomized controlled experiment, metformin was not found to reduce major cardiovascular events (27). The effect of metformin on the treatment of MI in real world studies is still controversial. In addition, most clinical trials examining the relationship between metformin and MI were small, and no studies based on MR exploring the causal effect of metformin therapy on the risk of MI were found during data review. Therefore, this study aimed to use MR theory to select SNPs related to metformin from GWAS database as IVs, so as to indirectly reveal the causal relationship between metformin and MI at different stages and locations from the genetic level. The preliminary results suggested that metformin has no beneficial protective effect on MI, and may even be a risk factor for MI, old MI, acute MI, and acute transmural MI of inferior wall.

MR analysis has been widely used in academic research, although the strength of evidence is not as strong as randomized controlled trials (RCT), it is not limited by ethical and experimental conditions. It is also less susceptible to potential confounders and reverse causality compared to observational studies (32). Therefore, MR analysis is considered to be a natural RCT study, and its results are credible (42, 43). All the IVs included in this study were screened by the PhenoScanner database, and the outcome data used were derived from 6 large GWAS studies. There was no obvious heterogeneity or pleiotropy among the IVs, so the analytical conclusions are robust.

This study also has some limitations: using GWAS data, it is impossible to explore any potential non-linear relationships or stratification effects created by age, gender, concomitant diseases and so on, which may bias the results; second, this study cannot verify whether the causal relationship between metformin treatment and MI will change with the dose or timing of metformin; finally, GWAS data only include people of European descent, and the conclusions are not representative of other ethnic groups.

In summary, from the genetic level, there is no obvious causal association between metformin and acute transmural MI of anterior wall, while for MI, old MI, acute MI, and acute transmural MI of inferior wall, it may be a risk factor. Combined with other RCT studies, it may still benefit from metformin in patients with diabetes and MI, while metformin may not be beneficial or even increase the risk of adverse effects in non-diabetic patients. In order to confirm the conclusion of this study, further standardized and large-sample clinical trials and related MR studies are still needed to further explore the potential effects and clinical significance of metformin in the treatment of MI.

## Data availability statement

The datasets presented in this study can be found in online repositories. The names of the repository/repositories and accession number(s) can be found in the article/**Supplementary Material**.

## Author contributions

YZ: Writing – original draft, Writing – review & editing, Data curation, Formal analysis, Software. XP: Writing – review & editing, Formal analysis. YC: Writing – review & editing, Data curation, Methodology. JS: Writing – original draft, Funding acquisition, Project administration.

## Supplementary material

Supplementary Table 1 Detailed information about 16 SNPs identified as IVs to assess the genetic association between metformin and outcomes of MI.

Supplementary Table 2 Detailed information about 84 SNPs identified as IVs to assess the genetic association between metformin and outcomes of old MI.

Supplementary Table 3 Detailed information about 39 SNPs identified as IVs to assess the genetic association between metformin and outcomes of acute MI.

Supplementary Table 4 Detailed information about 26 SNPs identified as IVs to assess the genetic association between metformin and outcomes of acute transmural MI of inferior wall.

Supplementary Table 5 Detailed information about 34 SNPs identified as IVs to assess the genetic association between metformin and outcomes of acute transmural MI of anterior wall.
